# A Parameter for IL-10 and TGF-ß Mediated Regulation of HIV-1 Specific T Cell Activation Provides Novel Information and Relates to Progression Markers

**DOI:** 10.1371/journal.pone.0085604

**Published:** 2014-01-09

**Authors:** Andreas Lind, Kristin Brekke, Frank Olav Pettersen, Tom Eirik Mollnes, Marius Trøseid, Dag Kvale

**Affiliations:** 1 Department of Infectious Diseases, Oslo University Hospital, Oslo, Norway; 2 Institute of Clinical Medicine, University of Oslo, Oslo, Norway; 3 Department of Immunology, Oslo University Hospital, Oslo, Norway; 4 Research Laboratory, Nordland Hospital, Bodø, and University of Tromsø, Tromsø, Norway; 5 K. G. Jebsen Inflammation Research Centre, University of Oslo, Oslo, Norway; University of Hawaii, United States of America

## Abstract

HIV replication is only partially controlled by HIV-specific activated effector T cells in chronic HIV infection and strategies are warranted to improve their efficacy. Chronic T cell activation is generally accompanied by regulation of antigen-specific T cell responses which may impair an effective control of chronic infections. The impact of HIV-induced T cell regulation on individual patients’ disease progression is largely unknown, since classical T cell activation assays reflect net activation with regulation as unknown contributing factor. We here explore a quantitative parameter for antigen-induced cytokine-mediated regulation (R_AC_) of HIV-specific effector T cell activation by functional antibody-blockade of IL-10 and transforming growth factor-β. HIV Env- and Gag-specific T cell activation and R_AC_ were estimated in peripheral blood mononuclear cells from 30 treatment-naïve asymptomatic HIV-infected progressors (CD4 count 472/µl, HIV RNA 37500 copies/ml) stimulated with overlapping peptide panels for 6 days. R_AC_ was estimated from differences in T cell activation between normal and blocked cultures, and related to annual CD4 loss, immune activation (CD38) and microbial translocation (plasma lipopolysaccharides). R_AC_ was heterogeneously distributed between individual patients and the two HIV antigens. Notably, R_AC_ did not correlate to corresponding classical activation. Env R_AC_ correlated with CD38 and CD4 loss rates (r> = 0.37, p = <0.046) whereas classical Gag activation tended to correlate with HIV RNA (r = −0.35, p = 0.06). 14 patients (47%) with low R_AC_’s to both Env and Gag had higher CD8 counts (p = 0.014) and trends towards lower annual CD4 loss (p = 0.056) and later start with antiretroviral treatment (p = 0.07) than the others. In contrast, patients with high R_AC_ to both Env and Gag (n = 8) had higher annual CD4 loss (p = 0.034) and lower CD8 counts (p = 0.014). R_AC_ to Env and Gag was not predicted by classical activation parameters and may thus provide additional information on HIV-specific immunity. R_AC_ and other assessments of regulation deserve further in-depth exploration.

## Introduction

Chronic human immunodeficiency virus type 1 (HIV) infection leads to a variable but progressive loss of immune functions in most patients. The progression rate is mainly influenced by two opposing factors, namely HIV-associated chronic immune activation [1]–[3] and the efficacy of HIV-specific T cell responses [4], [5]. Chronic immune activation expressed by CD38 on T cells, correlates strongly to disease progression and mortality [6]–[8]. It is partly sustained by enhanced systemic translocation of microbial products such as bacterial lipopolysaccharide (LPS) [9], [10] and induces polyclonal B and T cell activation [11], [12], accelerated T cell turnover [13], [14] and immune exhaustion [2], [15]. Effective viral control, on the other hand, seems to depend on the presence of polyfunctional HIV-specific CD8^+^ T cells [4].

A less clarified aspect of HIV-specific immunity is downregulation of the HIV-specific effector T cells, where regulatory T cells (Treg) play a central role [16], [17]. Regulation of effector T cells protects the host from damage in chronic infection, but may also impair effective immune control. It is mediated by a number of mechanisms, including the expression of inhibitory receptors in the immune synapse such as CTLA-4 [18] and programmed death-1 (PD-1) [19], [20], or via soluble inhibitory cytokines, particularly IL-10 and transforming growth factor-β (TGF-ß). These two key inhibitory cytokines impede pro-inflammatory responses by T cells, natural killer cells, monocytes and macrophages and are secreted by a number of cell types including Treg [21]–[24].

The efficacy of T cell responses depends on the sum of stimulatory and regulatory signals. T cell regulation has been intensively studied, but with focus on single regulating mechanisms. However, how these various regulating mechanisms finally and in concert influence HIV-specific T effector cells and disease progression in individual patients has been little explored. This might be assessed for T cells *in* *vitro* by blocking downstream intracellular regulatory signal pathways during antigen stimulation. Recently we tested such an in vitro *quantitative* parameter for regulation in patients on antiretroviral treatment (ART) during reboost with a Gag peptide-based therapeutic HIV vaccine [25]. We estimated vaccine-specific cytokine-mediated regulation of CD8^+^ T cell responses by blocking the effects of IL-10 and TGF (antigen-induced cytokine-mediated regulation, R_AC_). Notably, changes in R_AC_ explained the substantial variations in booster efficacy, including cases where vaccine responses waned after each booster.

Since R_AC_ seemed to reflect important features of HIV vaccine-specific T cell immunity during immunization, we hypothesized that the same parameter also would provide novel information in natural chronic HIV infection. In this study, we therefore compared R_AC_ and activation of Gag- and Env-specific T effector cells in treatment-naïve patients. We found R_AC_ to be heterogeneous, both between individual patients and between the two HIV antigens, and unfavourably related to HIV progression.

## Materials and Methods

### Patients

Thirty asymptomatic HIV-1 seropositive ART-naïve viremic progressors were included (23 males, 7 females). Their clinical characteristics are shown in Table 1. The patients represented a spectre of HIV-associated immune activation determined by CD38 densities on total CD8^+^ and CD8^+^PD-1^+^ T cells [8] and were chosen from a larger cross-sectional prospective study on immunological factors in HIV. The study was approved by the Norwegian South-Eastern Regional Committee for Medical and Health Research Ethics. Informed consent was signed by each participant.

**Table 1:** Cohort characteristics.

	All (n = 30)
	Median (IQ range)
Age (years)	42 (33–49)
Time HIV seropositive (months)	57 (16–83)
CD4+ T cell count (×10^6^/l)	472 (325–695)
CD8+ T cell count (×10^6^/l)	1084 (788–1828)
HIV-RNA in plasma (copies/ml)	37500 (2300–72000)
Annual CD4 T cell count loss (cells ×10^6^/l)	11 (−69–177)
β_2_-microglobulin in serum (mg/l)	2.5 (1.9–3.3)
CD38 on CD8+ T cells (molecules/cell)	3285 (1834–7226)
CD38 on CD8+CD38+PD-1+ T cells (molecules/cell)	4127 (2095–8704)
LPS (pg/µl)	70 (59–86)

### Activation Assays and Flow Cytometry

Peripheral-blood mononuclear cells (PBMC) were isolated using Cell Preparation Tubes (Becton Dickinson (BD), CA, USA) and preserved, thawed and cultured in serum-free AIM culture medium containing 0.5% human albumin at 5% CO2 at 37°C, as described elsewhere [26]. To evaluate activation and proliferation parameters, the fractions of T cells co-expressing CD25 and HLA-DR [27] or having low carboxyfluorescein succinimidyl ester (CFSE^dim^) signal [28] were compared. PBMC were pulse-labelled with CFSE (3 µM, 5 min (Invitrogen Molecular Probes, OR, USA) as detailed previously [29] and subjected to HIV antigens (four HIV-1 Gag p24 consensus peptide sequences, represented by 15-mer overlapping by 2 amino acid panels [29] and non-HIV antigens (23 15-mer peptides from cytomegalovirus, Epstein-Barr virus and influenza virus (CEF, Mabtech, Sweden)).

For the estimates of antigen-specific cytokine-mediated regulation of T cell activation (R_AC_), cryopreserved PBMC were thawed, washed and reconstituted in serum-free AIM overnight, and then stimulated with complete 15-mer Env or Gag overlapping peptide panels (NIH AIDS Research and Reference Reagent Program, MD, USA) as detailed elsewhere [30]. Peptide panels in all experiments were used at 2 µg/ml/peptide. Peptide-exposed and control cultures were in parallel incubated with inhibitory monoclonal antibodies (mAbs) to IL-10 and TGF-ß, each at 10 µg/ml final concentration according to the instructions by the manufacturer (R&D Systems Europe, Abingdon, UK), a concentration that abolished IL-10 in cell culture supernatants (Luminex assay of supernatants from antigen stimulated T cells cultured for 6 days, data not shown). Staphylococcal enterotoxin B (Sigma-Aldrich, MO, USA) was used as positive control at 0.5 µg/ml.

Cells were cultured at 37 C^o^ in 5% CO_2_ for 6 days, and then harvested, stained and prepared for flow cytometric analysis as previously described [31]. The following fluorochrome-labelled mAbs were used: CD3 Pacific Blue, CD8 AmCyan, HLA-DR PE-Cy7 (BD), CD4 PE and CD25 APC (eBioscience, CA, USA). 7-aminoactinomycin (7-AAD, BD) was added to discriminate between viable and non-viable cells according to the manufacturer. Flow cytometry data were obtained with a BD FACS Canto II with BD Diva software v6.1. Only lymphocyte and lymphoblast gates containing live 7-AAD^−^ CD3^+^ T lymphocytes were evaluated.

### Quantification of Env and Gag Related T Cell Activation

**Figure 1:**
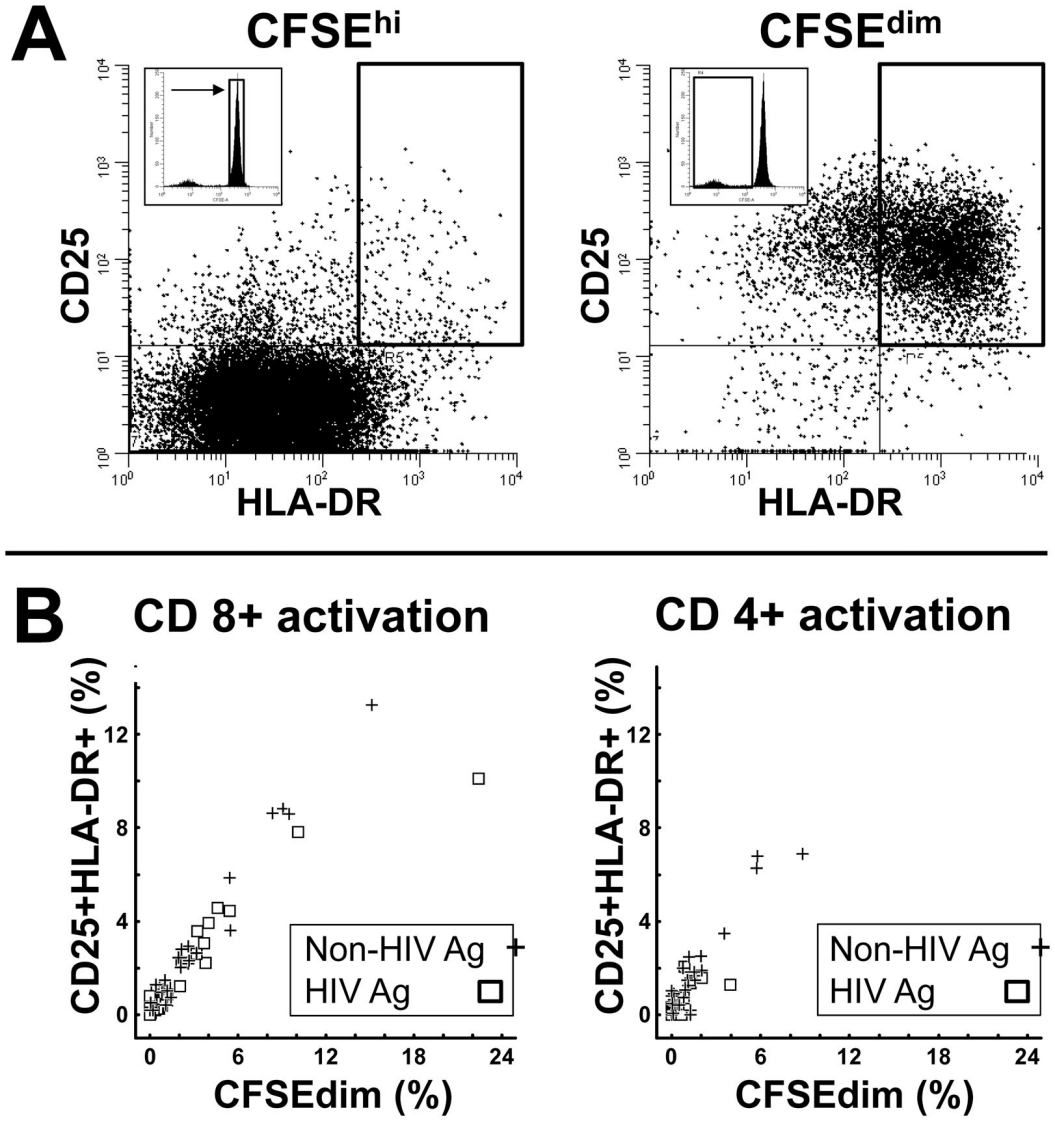
Comparison of CD25^+^HLA-DR^+^ expression and CFSE^dim^ as measures for antigen-induced activation and regulation of T cells. CFSE-labelled PBMC from 28 HIV-infected individuals were stimulated for 6 days with peptides encoding either HIV p24 consensus regions (HIV Ag) or a pool of commonly encountered non-HIV viral peptides (Non-HIV Ag). **A.** Co-expression of CD25 and HLA-DR on live CD8^+^CD3^+^ T cells on non-divided CFSE^high^ (left panel) and proliferated CFSE^dim^ T cells (right panel), showing excessive difference in fractions of CD25^+^HLA-DR^+^ in the activated subset to the right. **B.** Scatter plots of activation measured in the same culture by CFSE^dim^ or HLA-DR^+^CD25^+^, respectively, to non-HIV (**+**) and HIV antigen (□) within the CD8^+^ (left panel) and CD4^+^ (right panel) T cell subsets. Significant and high correlations obtained for both Non-HIV antigens (CD8^+^, r = 0.92, p<0.001: CD4^+^ r = 0.64, p<0.001) and HIV Gag p24 (CD8^+^, r = 0.90, p<0.001: CD4^+^ r = 0.71, p<0.001).

Antigen-specific activation of T cell subsets was defined as the difference in activation marker between peptide-stimulated cells and corresponding control cells without peptides. In preceding experiments exploring T cell regulation by HIV vaccine antigens in patients on ART, regulation and activation were determined by differences in proliferation (CFSE^dim^) in CFSE pulse-labelled cells [25]. In our experience, thawed PBMC samples from ART-naïve individuals are more vulnerable to toxic effects of CFSE [32], even after short exposure and low concentrations. We therefore compared fractions of CFSE^dim^, defined by median fluorescence intensities equal to or below the second proliferated generation in CFSE-labelled PBMC, and fractions of CD25^+^HLA-DR^+^. These parameters reflect overlapping aspects of T cell activation [27], i.e. proliferation, IL-2 receptor expression and increased HLA class II expression), as illustrated in Fig. 1A, with correlating activation results after exposure to both non-HIV and HIV antigens (Fig. 1B). T cell activation within the cohort was therefore determined by the frequency of subsets co-expressing CD25 and HLA-DR in antigen-stimulated cultures corrected for unstimulated controls [27].

### Quantification of Env- and Gag-induced Cytokine-mediated T Cell Regulation

**Figure 2:**
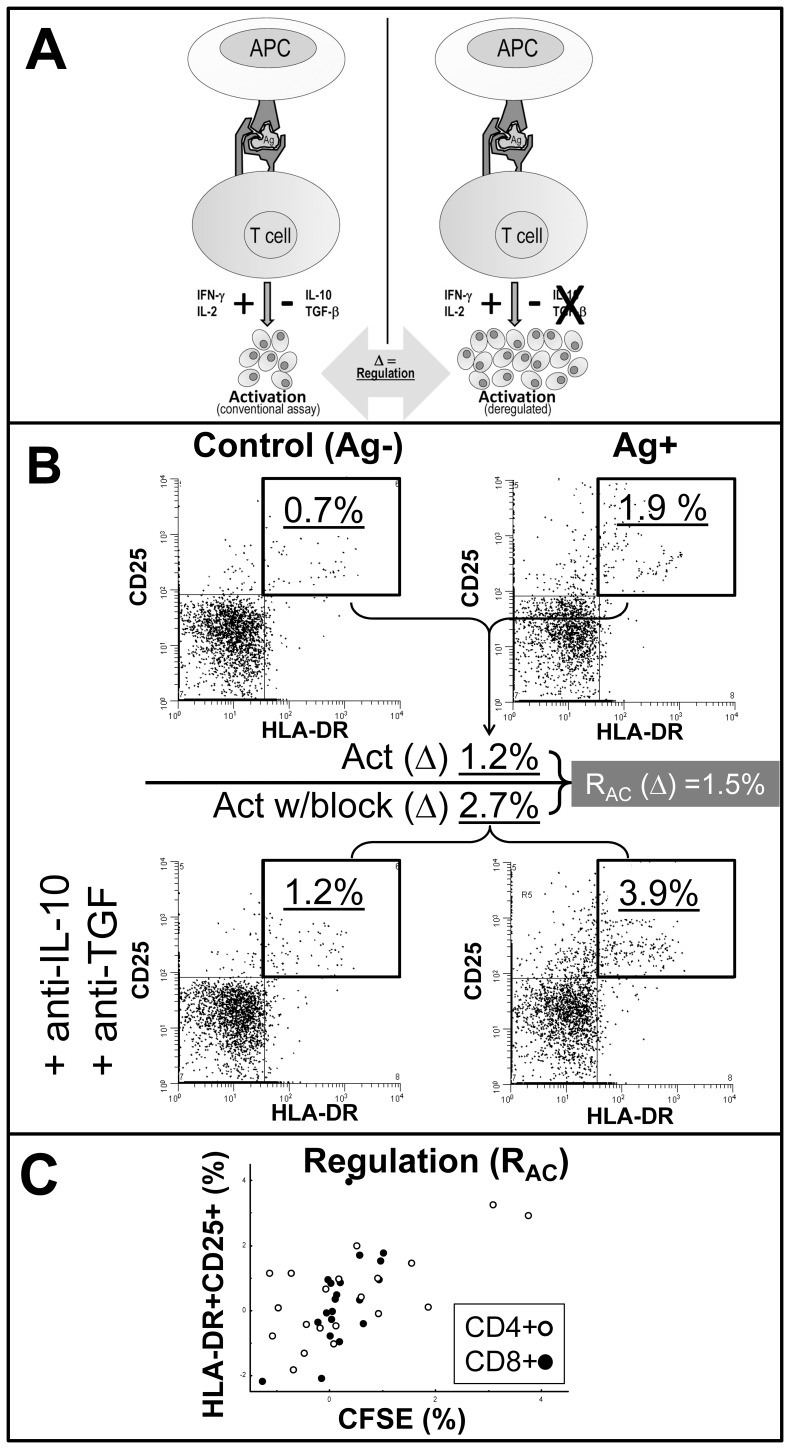
Schematic outline and examples of the T cell regulation assay. **A.** Schematic outline of the assay measuring antigen-induced cytokine-mediated induced regulation (R_AC_) of T cell activation by IL-10 and TGF-ß. Left panel shows a conventional T cell assay where the final activation measurements are regarded as net results of proinflammatory and regulatory signals. Right panel show a possible outcome when blocking T cell regulatory cytokines. T cell regulation (**Δ**) is here defined as the difference in activation responses to the same antigen between these panels. **B.** Detailed example calculating classical activation (upper panels) and regulation (calculated from activation in the presence blocking mAbs to IL-10 and TGF, lower panels), all gated for viable CD8^+^ CD3^+^ T cells. Activation in both upper and lower panels calculated as the difference (**Δ**) in CD25^+^HLA-DR^+^ fractions between control and antigen-stimulated (Ag^+^). Note a typical slight increase in background activation (lower left panel). Regulation (R_AC_) calculated as the difference (**Δ**) between activation in cytokine-blocked culture and conventional activation culture. **C.** Scatter plots of regulation calculated in the same cultures by CFSE^dim^ or HLA-DR^+^CD25^+^, respectively, for the CD8^+^ (λ, r = 0.67, p<0.001) and CD4^+^ (Υ, r = 0.45, p = 0.026) T cell subsets.

In parallel with classical activation cultures, IL-10 and TGF-ß blocked activation was determined as the difference between antigen-stimulated and control samples that received IL-10 and TGF-ß blocking mAbs (Fig. 2A). The magnitude of antigen-induced IL-10 and TGF-ß mediated regulation of T cell activation (R_AC_) was calculated by the difference in activation between these two culture conditions (Fig. 2B). R_AC_ calculated by CFSE^dim^ correlated with R_AC_ determined by the CD25^+^HLA-DR^+^ subsets (Fig. 2C).

### LPS Measurement

EDTA plasma obtained concurrently with the isolation of PBMC was separated and snap-frozen at −70°C. LPS was later measured en bloc in thawed plasma with the Limulus Amebocyte Lysate chromogenic assay (Lonza, MD, USA) according to the manufacturer’s instructions with the following modifications: Samples were diluted 10-fold to avoid interference with background colour and preheated to 70°C for 12 minutes prior to analysis to dissolve immune complexes, as previously described [10].

### Plasma Levels of Cytokines and Chemokines

Soluble cytokines representing Th1 (TNF-α, INF-γ) and Th2 (IL-4, IL-5, IL-10 and IL-13) profiles were measured in snap-frozen EDTA plasma (see above) using Bio-Plex XMap technology (TX, USA) with a Luminex IS100 instrument (BIO-RAD, CA, USA) and Bio-Plex manager Software v6, according to the instructions by the manufacturer.

### Statistics

To not underestimate regulation, antigen-specific activation readouts relative to control cultures were treated as raw data. Non-parametrical statistics were applied throughout the study; Mann-Whitney U- and Kruskal-Wallis test to compare differences between two or more groups, and Spearman Rank for correlation analysis. All continuous variables are presented as medians (interquartile range). The Fisher Exact test was performed to analyse cross-tabulated categorical data. The annual CD4 count loss rates were calculated as previously described [26]. Statistica v7 statistical software (StatSoft Inc., OK, USA) was used for all analysis. A p-value ≤0.05 was regarded as significant.

## Results

### Cohort Characteristics Including Parameters for Immune Activation

Thirty asymptomatic ART-naïve HIV-infected patients (CD4^+^ T cell counts, 472; HIV RNA, 37,500 copies/ml, medians) were included to represent a spectrum of HIV-associated immune activation. CD38, microbial translocation (LPS) and HIV RNA correlated (r between 0.44–0.60, p<0.02, detailed data not shown). In keeping with previous observations where CD38 density on CD8^+^ T cells and on CD8^+^PD-1^+^ cells had higher correlation with other progression markers than frequencies of CD38^+^HLA-DR^+^CD8^+^ T cells [8], [25], [26], CD38 density was used to represent chronic immune activation in the following analysis.

### T cell Activation by Gag and Env

**Figure 3:**
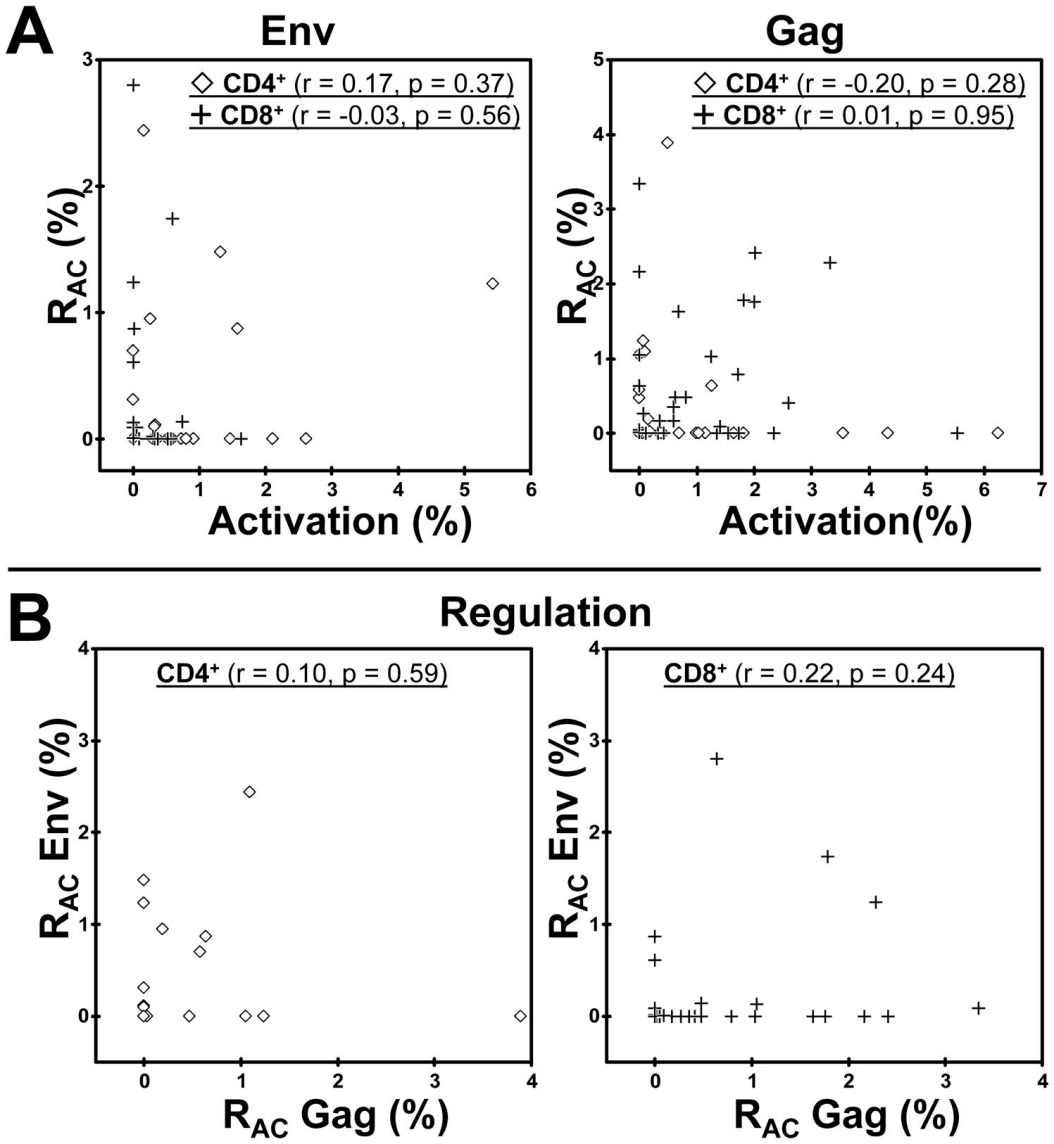
Insignificant relations between T cell activation and R_AC_ induced by Env and Gag peptide panels. **A.** Relations between regulation and activation to Env (left panels) and Gag (right panels) within the CD4^+^ and CD8^+^ T cell subsets respectively, correlation coefficients indicated. **B.** Relations between Env- and Gag-induced regulation within the CD4^+^ (right panel) and the CD8^+^ (right panel) T cell subsets, respectively, correlation coefficients indicated.

T cell activation to Gag and Env peptide panels varied between patients and was generally higher for Gag, in keeping with previous observations [30] (Fig. 3A, x-axis). Moreover, Gag and Env activation correlated within both the CD8^+^ (r = 0.40, p = 0.027) and CD4^+^ (r = 0.53, p = 0.003) T cell subsets (data not shown).

### Variable T Cell Regulation (R_AC_) without Correlation to Activation

A parameter for HIV antigen-specific cytokine-mediated T cell regulation (R_AC_) was determined by parallel antigen activation cultures and controls in the absence and presence of IL-10 and TGF-ß blocking mAbs. It should be noted that R_AC_ calculated by CFSE correlated significantly with R_AC_ determined by the co-expression of CD25 and HLA-DR (Fig. 2C).

A substantial variability was observed in R_AC_ related to Gag and Env exposure (Fig. 3A, y-axis). No correlations were found between R_AC_ induced by the two HIV antigens (Fig. 3B), in contrast to the corresponding activation. Perhaps more importantly, Gag or Env related R_AC_ and corresponding activation did not correlate (Fig. 3A). Thus, R_AC_ quantified this way could not have been predicted by the conventional activation assay.

### Activation and R_AC_ to HIV-antigens in Relation to Progression Markers

**Figure 4:**
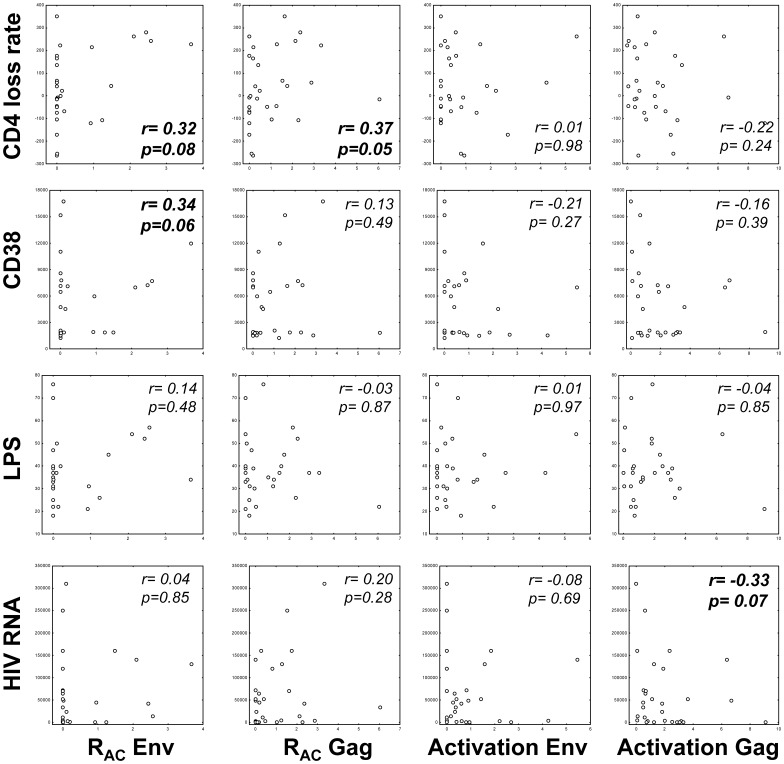
Relations between Env- and Gag-induced R_AC_ and activation and markers related to progression of chronic HIV infection. R_AC_ and corresponding activation (x-axis), for simplicity represented as overall CD3+ estimates (CD4^+^ plus CD8^+^ combined) induced by Env and Gag peptide panels, respectively, and in relations to various progression markers (y-axis). Spearman rank r and corresponding p-values indicated, values with p<0.10 bolded.

We next explored how R_AC_ was related to markers of chronic HIV activation (CD38 density on CD8+ T cells and PD-1 subsets [8], [26]), microbial translocation (LPS), HIV replication and annual CD4^+^ T cell loss rates. Significant and unfavourable correlations were revealed between Env related R_AC_ in either T cell subsets and chronic immune activation (CD8+, r = 0.41, p = 0.024) and CD4 loss rates (CD4+, r = 0.39, p = 0.032), whereas Gag-induced T cell activation tended to correlate with HIV RNA (CD8+, r = −0.35, p = 0.060). These heterogeneous relations are depicted in Fig. 4, for simplicity illustrated by overall CD3^+^ T cell activation and regulation.

### Clusters of Patients with Low and High HIV Antigen-induced Regulation

**Figure 5:**
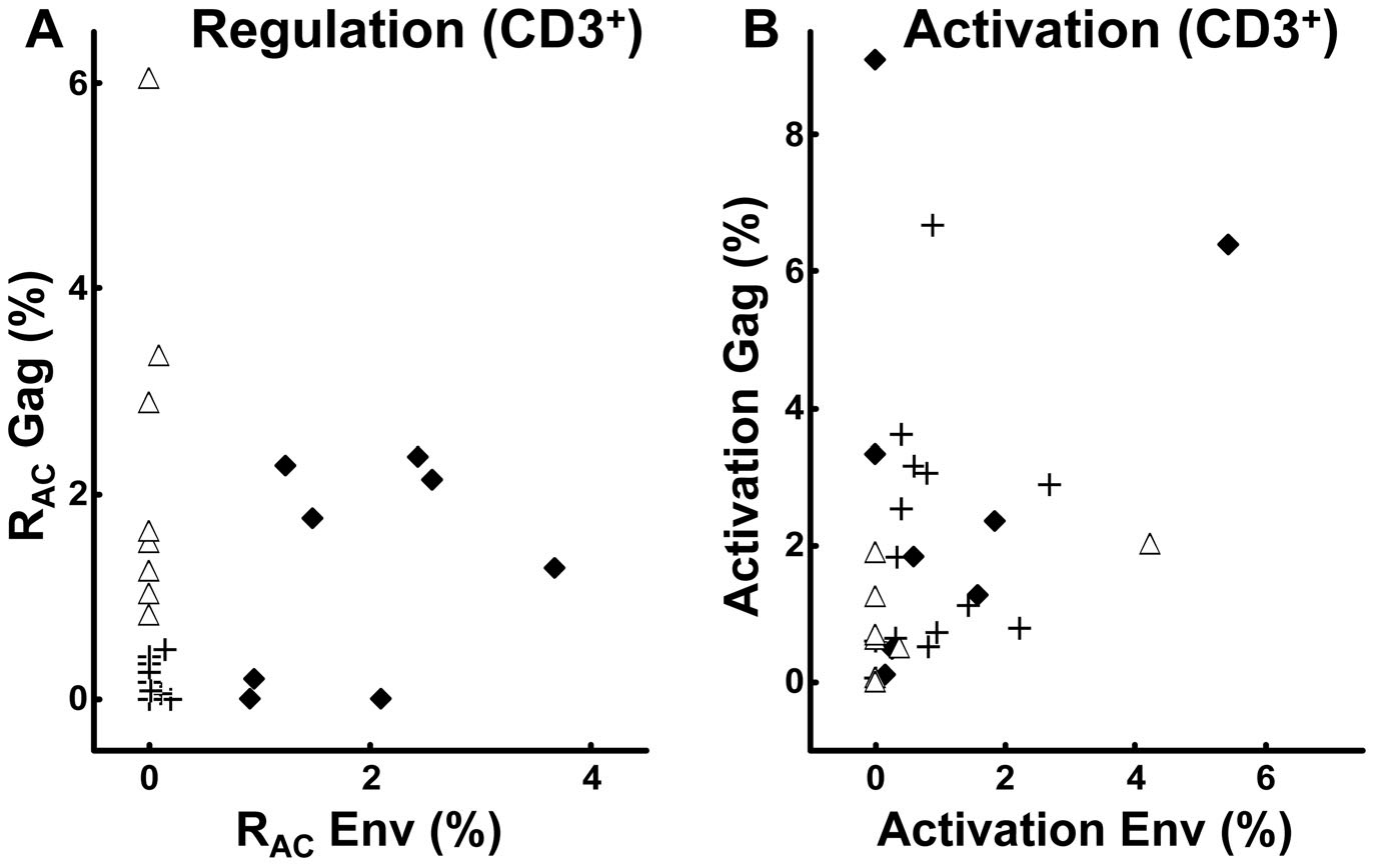
Relations between total CD3^+^ T cell activation and R_AC_ induced by Env and Gag peptide panels. **A.** Relations between total CD3^+^ T cell regulation calculated after Env and Gag peptide panel stimulations, respectively. Low regulators (**+**), Gag regulators (▵) and Pan regulators (⧫) indicated as defined in the text. **B.** Relations between total CD3^+^ T cell activation to Env and Gag, respectively, in the same patient groups as in panel A.

One cluster of patients appeared to have low R_AC_ induced by both Gag and Env within the CD4^+^ and CD8^+^ subsets (Fig. 3B). The same cluster was seen when we examined R_AC_ for all CD3^+^ T cells (Fig. 5A). This is in keeping with the notion that IL-10 and TGF-ß inhibit both the CD4^+^ and CD8^+^ T cell subsets [23], [33]. This cluster of patients with overall low R_AC_ induced by Gag and Env was defined as *Low regulators* [n = 14 (47%)] (Fig. 5A) whereas the remaining 53% (n = 16) were termed *High regulators*. Notably, the magnitude of R_AC_ in suppressing corresponding activation was quite substantial for the High regulator patients, as illustrated by high R_AC_/Activation-ratios [3.0 (0.8–4.1) for Env and 2.4 (0.8–12.4) for Gag, respectively] (data not shown). Again, conventional activation for CD3^+^ T cells did not correlate with the corresponding R_AC._ Thus, High regulators could not have been identified by the activation assay (Fig. 5B).

**Figure 6:**
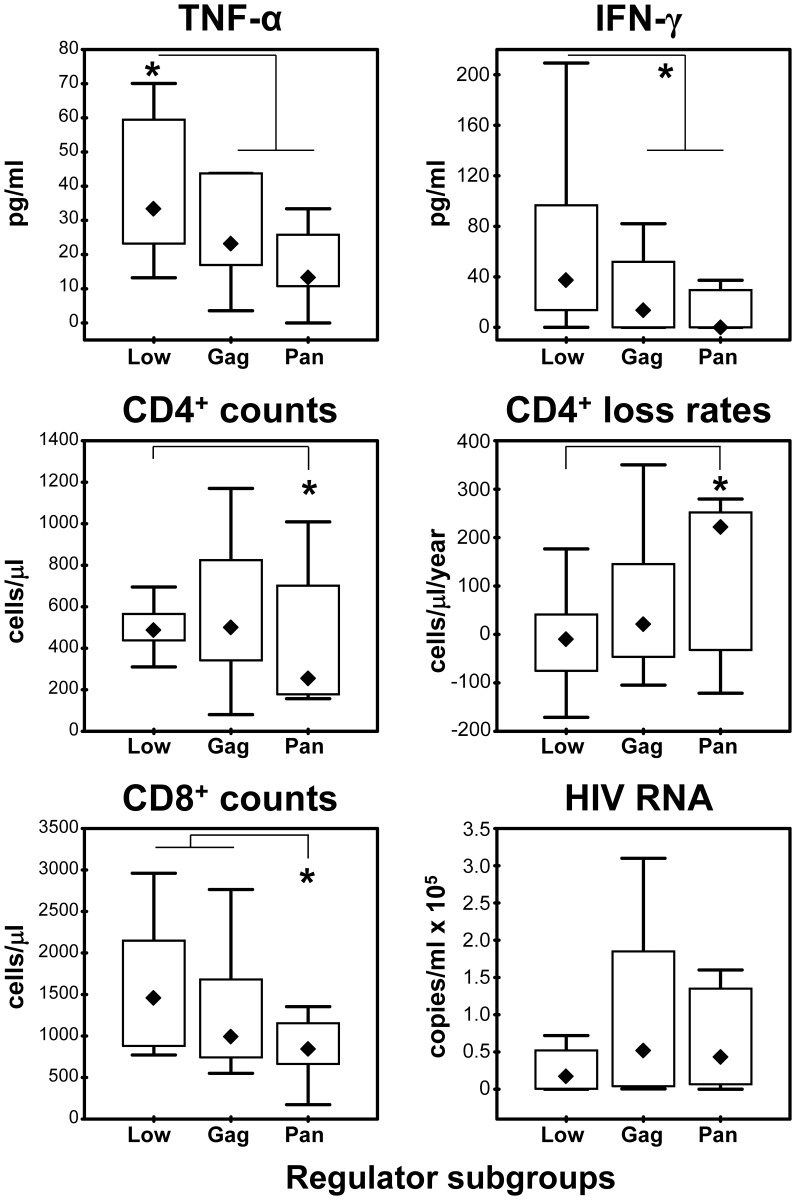
Distributions between regulator subgroups. Box and whisker plots representing medians, interquartile ranges and overall ranges for cytokines in snap-frozen plasma (upper two panels), CD4 counts and CD4 loss rates (two middle panels) and CD8 counts as well as HIV RNA levels (two lower panels). “Low regulators” (Low) as defined in the text are represented as one group, whereas the “High regulator” patients are split into “Gag regulators” (Gag) and “Pan regulators” (Pan), respectively. Significant differences p<0.05 between groups (Mann-Whitney) indicated (*).

The Low and High regulator patient groups were also compared with respect to clinical parameters, immune activation, LPS and conventional activation. High regulators had lower CD8 counts in blood (p = 0.031) and a trend towards faster CD4 loss rates (p = 0.056) (Table 2). High regulators also had significantly lower levels of plasma Th1 cytokines INF-γ (p = 0.04) and TNF-α (p = 0.04) (Fig. 6), but no differences were found for Th2 cytokines including IL-10 between the two regulation groups.

**Table 2:** Characteristics of regulator groups.

	Low regulators (n = 14)	High regulators (n = 16)	Low vs. high regulators
	Median (IQ range)	Median (IQ range)	p*
Age (years)	43 (39–49)	40 (30–52)	0.755
Time HIV seropositive (months)	65 (16–100)	52 (21–68)	0.510
CD4+ T cell count (×10^6^/l)	488 (438–565)	392 (225–825)	0.262
CD8+ T cell count (×10^6^/l)	1458 (880–2148)	938 (663–1261)	**0.031**
HIV-RNA in plasma (copies/ml)	17000 (670–52000)	43000 (4000–135000)	0.220
Annual CD4 T cell count loss (cells ×10^6^/l)	−10 (−75–41)	62 (−46–235)	*0.056*
β_2_-microglobulin in serum (mg/l)	2.5 (2.1–3.0)	2.3 (1.5–5.3)	0.965
CD38 on CD8+ T cells (molecules/cell)	1894 (1770–7116)	4271 (1688–11165)	0.244
CD38 on CD8+CD38+PD-1+ T cells (molecules/cell)	2541 (1938–8101)	4946 (1962–13798)	0.228
LPS (pg/µl)	70 (57–76)	70 (59–92)	0.693

Comparisons between patient groups, p<0.05 bolded, p<0.10 italic.

### Characterization of Study Patients with High HIV Antigen-induced Regulation

Examining the High regulator patients in more detail, we found that they either had substantial R_AC_ induced by Gag (denoted *Gag regulators*, n = 8), or by both Gag and Env (*Pan regulators*, n = 8) (Fig. 5A). Gag regulators appeared more similar to Low regulators in most parameters except that they had less conventional activation to both Gag (CD4^+^ subset, p = 0.016) and Env (p = 0.025). Pan regulators, on the other hand, had a profile compatible with more accelerated disease, such as higher annual CD4 loss (221 vs −10 cells/year, p = 0.034), lower CD8 counts (median 841 vs 1458 cells/µl, p = 0.014) and possibly lower CD4 counts (median 254 vs 488 cells/µl, p = 0.065) compared with the Low regulator patients (Fig. 6). Thus, one might speculate whether Gag and Pan regulators represent a continuum of an unfavourable regulator phenotype which could not be identified by the classical activation assay. Finally, of the 14 patients who had started ART according to current guidelines within one year post-inclusion, more patients tended to be Pan regulators than belonging to the other subgroups (6 of 8 vs. 8 of 22, p = 0.07).

## Discussion

HIV-specific T effector cells are potentially able to control viral replication in HIV infection, but their responses are critically weakened by the initial loss of HIV-specific CD4^+^ T cells, viral immune escape, and T cell exhaustion driven by immune activation [5], [34]. An additional counteracting factor might be the regulation of effective HIV specific T effector cells. We here assessed a functional *quantitative* parameter for T cell regulation (R_AC_) which we think could be relevant when evaluating HIV infected patients and developing therapeutic vaccines. Therapeutic vaccines might play an essential role in a future cure for HIV by inducing effective T cell responses against re-activated, latently infected cells [34]. Theoretically, pre-existing or induced regulation can evoke T cell anergy and thus hamper the effects of therapeutic vaccination in some patients. This notion was supported by our recent observation where changes in R_AC_ explained variable and in some cases negative responses to therapeutic HIV vaccine boosters [25].

To our knowledge, this is the first attempt to determine R_AC_ or similar quantitative parameters for HIV antigen-specific regulation in chronically infected treatment-naïve patients. The study was motivated by our expectation that R_AC_ would provide additional prognostic information. We found considerable variability in R_AC_ not only between individual patients, but also between the two tested HIV antigens. Thus, our data suggest that at least in some patients, R_AC_ does not reflect “global” regulation of HIV antigens. R_AC_ was in some cases substantial, exceeding activation more than ten-fold. Moreover, R_AC_ did not relate to corresponding conventional activation readouts, showing that it provided additional otherwise hidden information.

This exploratory approach to characterize a parameter apparently reflected at least some aspects of cytokine-mediated regulatory “capacity” in the individual patient. However, although our data suggest that R_AC_ can differentiate HIV-infected patients in a new way and may reflect processes that are related to progression of HIV, our choices of assay read-out and culture conditions needs to be commented: Several assays are frequently used to assess HIV-specific T cell activation and function. For example, polyfunctional T cells in 6 to 18 h cultures have been shown to coincide with control of viral replication [32]. However, we did not prioritize this assay due to shortage of cells from this clinically well-defined cohort, and 6 day cultures were chosen for several reasons: First, we expected *a priori* that antigen-related regulation is a slower, secondary response, to primary activation. This assumption is in keeping with the observation that stimulation of resting Treg reach maximal expression of FoxP3 34–44 h after simulation [35]. Second, it is still not clear whether early polyfunctionality actually persists over time, including early markers for proliferation such as Ki-67 [32]. Third, a fundamental element of effector lymphocytes is the ability to proliferate, indicating responsiveness to IL-2 (via its receptor CD25), whereas proinflammatory cytokines such as IFN-γ upregulate HLA class II (including DR) on T cells. Moreover, HIV-specific proliferative T cell responses have been long known to associate with slow progression [36]. Our assay use changes in CD25+HLA-DR+ as readout, parameters that both reflect activation and proliferation, the latter illustrated in Fig. 1A. Nevertheless, we appreciate that our approach only reflect one out of several ways by which classical “net” T cell responses can be estimated *in vitro*. Indeed, other major regulatory pathways may influence overall activation. Finally, in-depth interpretation and characterization of our assay can certainly be extended, such as to address whether the “gain” in activation by blockade of regulatory pathways also provides an increase in effector cell functions, such as cytotoxic capacity or polyfunctionality.

A possible clinical relevance of this new exploratory parameter was suggested by the significant correlations between R_AC_ and the classical prognostic markers CD38 and CD4 loss rates. These correlations were not found for the activation results (Fig. 4). Even if the study included only a limited number of cases, we were still able to cover a wide spectrum of chronic immune activation. Gag-specific T cell responses correlated negatively with concurrent HIV RNA levels, an association also found in other and larger study cohorts [37], [38]. It should be noted that our group favours bead-calibrated measures for CD38 density rather than the more simple and conventional measure for HIV-associated chronic immune activation, namely % CD38^+^HLA-DR^+^. We have previously shown that CD38 density is even better related to other progression markers [8], [25], [26].

Post-hoc we observed clusters of patients having either particularly low (Low regulators) or high (High regulators) regulation (i.e. R_AC_). The High regulators seemed to have more rapid HIV progression, in keeping with our expectation. In contrast, Low regulators had more favourable clinical characteristics in terms of slower CD4 loss rates and higher CD8 counts [39]. The levels of the proinflammatory cytokines TNF-α and IFN-γ were also higher in Low regulators. This has previously been interpreted as a sign of unfavourable immune activation in patients with lower CD4 counts [40], [41]. From our data, derived from patients with higher CD4 counts, one might conversely speculate whether higher TNF-α and IFN-γ levels rather reflect a beneficial type of immune activation.

R_AC_ or similar quantitative parameters for HIV antigen-specific regulation should be further explored in larger cohorts. This may help to better understand the complex interplay between regulation and activation, to select patients for immune therapy studies, and to determine the prognostic significance of regulation. Future studies should also explore the individual contribution of IL-10 and TGF-ß along with other regulating mechanisms such as CTLA-4 and PD-1. This was hampered by a scarcity of patients and samples in this study. Both a broader range of HIV antigens and even non-HIV antigens should be tested. In this study Gag was selected based on the relation between Gag-specific T cell responses to control viral replication [37], [38] and Env as a relevant antigen for HIV vaccines.

## Conclusions

In summary, this study on regulation of Gag- and Env-specific T cell activation by IL-10 and TGF-ß (R_AC_) in chronic HIV infection revealed heterogeneous levels of regulation between both patients and HIV antigens. The magnitude of R_AC_ was substantial in some individuals and R_AC_ could not be predicted by the corresponding, classical antigen-specific activation parameters. High R_AC_ seemed clinically unfavourable, particularly when induced by Env peptides. Thus, assessments of regulation deserve further in-depth exploration and extension to larger cohorts.
